# Vascular Injury Post Stent Implantation: Different Gene Expression Modulation in Human Umbilical Vein Endothelial Cells (HUVECs) Model

**DOI:** 10.1371/journal.pone.0090213

**Published:** 2014-02-26

**Authors:** Jonica Campolo, Federico Vozzi, Silvana Penco, Lorena Cozzi, Raffaele Caruso, Claudio Domenici, Arti Ahluwalia, Michela Rial, Paolo Marraccini, Oberdan Parodi

**Affiliations:** 1 CNR Institute of Clinical Physiology, Milan and Pisa, Italy; 2 Department of Laboratory Medicine, Medical Genetics, Niguarda Ca' Granda Hospital Milan, Italy; 3 Interdepartmental Research Centre “E. Piaggio”, University of Pisa, Pisa, Italy; University Hospital Medical Centre, Germany

## Abstract

To explore whether stent procedure may influence transcriptional response of endothelium, we applied different physical (flow changes) and/or mechanical (stent application) stimuli to human endothelial cells in a laminar flow bioreactor (LFB) system. Gene expression analysis was then evaluated in each experimental condition. Human umbilical vein endothelial cells (HUVECs) were submitted to low and physiological (1 and 10 dyne/cm^2^) shear stress in absence (AS) or presence (PS) of stent positioning in a LFB system for 24 h. Different expressed genes, coming from Affymetrix results, were identified based on one-way ANOVA analysis with p values <0.01 and a fold changed >3 in modulus. Low shear stress was compared with physiological one in AS and PS conditions. Two major groups include 32 probes commonly expressed in both 1AS versus 10AS and 1PS versus 10PS comparison, and 115 probes consisting of 83 in addition to the previous 32, expressed only in 1PS versus 10PS comparison. Genes related to cytoskeleton, extracellular matrix, and cholesterol transport/metabolism are differently regulated in 1PS versus 10PS condition. Inflammatory and apoptotic mediators seems to be, instead, closely modulated by changes in flow (1 versus 10), independently of stent application. Low shear stress together with stent procedure are the experimental conditions that mainly modulate the highest number of genes in our human endothelial model. Those genes belong to pathways specifically involved in the endothelial dysfunction.

## Introduction

Endothelial cells (EC) are steadily exposed to a range of physical and biomechanical stimuli. Three primary hemodynamic forces act on endothelium: a) the radial pressure created by the hydrostatic blood forces, b) circumferential stretch or tension resulting by intercellular connections between the endothelial cells, and c) shear stress, the frictional force created by blood flow. These forces have been shown to induce several cellular events; in particular the presence of low shear, non-laminar flow is able to induce changes in gene expression profile that pre-dispose the endothelium to the initiation and development of atherosclerotic lesions [1]–[3]. Non-laminar flow promotes changes to endothelial gene expression, cytoskeletal arrangement, wound repair, leukocyte adhesion as well as to the vasoreactive, oxidative and inflammatory states of the artery wall. Disturbed shear stress also influences the site selectivity of atherosclerotic plaque formation as well as its associated vessel wall remodelling, which can affect plaque vulnerability.

Recent studies have highlighted that the placement of a stent against the artery wall may affect the arterial mechanical environment in very profound way [4], [5]. Stent application may directly injured endothelium through a mechanical stretching action that produces endothelial damage and denudation. Moreover, changes in flow patterns after stent positioning have been observed in experimental/computational flow study [5] and include large-scale vortex formation and strut-spacing dependent flow stagnation. The low shear stresses associated with flow stagnation could likely induce, together with endothelial damage, vascular changes that are responsible of intimal hyperplasia, a leading cause of restenosis which occurs in 20–30% of patients within 6–12 months after primary stenting [6].

Although several groups have reported that low shear stress compared to physiological one may affect gene expression profile of endothelial cells in different experimental systems [7]–[9], it is still unclear whether an invasive intervention like stent procedure may influence the transcriptional response of endothelium.

To study the simultaneous effects of both changes in shear stress and stent application on endothelial gene expression, we have developed an experimental model of laminar flow bioreactor (LFB) system with human cultured endothelial cells exposed or not exposed to stent procedure. RNA expression from different experimental conditions has been evaluated through the Affymetrix platform.

## Materials and Methods

**Figure 1:**
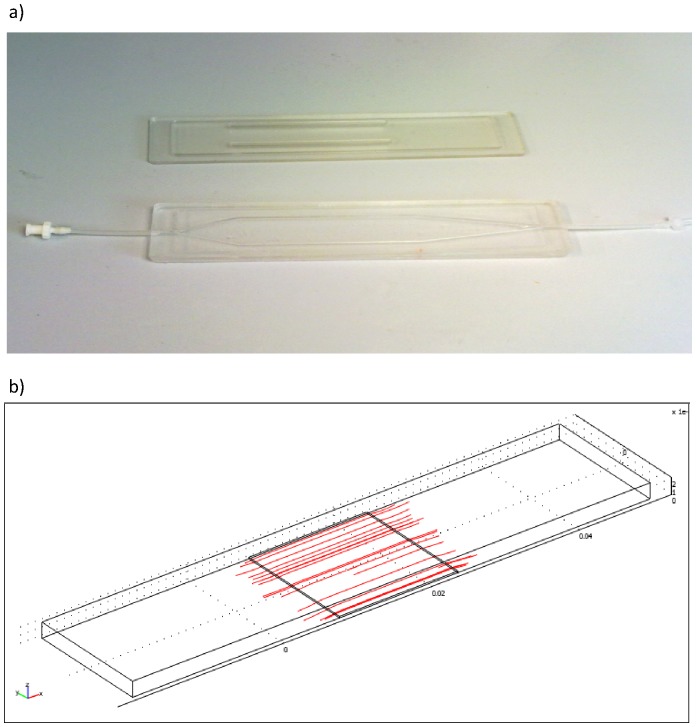
Flow chamber. The flow chamber is realized in polydimethylsiloxane and designed to enable cells to be subject to a large range of shear stress (a). The geometrical configuration has been modified to obtain an optimal laminar flow in the central zone (b) of the chamber.

We used a bioreactor system, designed and realized at Interdepartmental Research Centre “E. Piaggio” [10], that is a “natural” evolution of parallel and cone-plate systems but with a high uniformity in terms of shear stress. The geometrical configuration of flow chamber realized in polydimethylsiloxane, a silicone biocompatible polymer, has been modified to obtain an optimal laminar flow in the central zone (active region) of the cell chamber (Figure 1). Its particular shape was obtained after modelling analysis performed with finite element software for simulation of fluid dynamic flow (Fluent®). With this geometry, a central region with laminar flow and high wall shear stress values is obtained, which allows for simulating different regions of the cardiovascular system by adjusting flow rates.

For the *in vitro* stent experiments, we used a Crome-Cobalt bare metal stent ST 516 model (dimensions 2×1 cm) without any eluting drug (CID Saluggia, Italy).

### Endothelial cell culture

Fresh human umbilical cords were recovered from healthy females at the Obstetrics and Gynecology Unit of the Azienda Ospedaliera Universitaria Pisana, after obtaining written informed consent for use of these samples in research approved by the Local Ethics Committee of Area Vasta Nord Ovest. The umbilical cords were stored in PBS at 4°C, sent to our laboratory within 1 hour of delivery and treated anonymously conforming with the principles outlined in the Declaration of Helsinki. Umbilical vein was cannulated, washed with PBS solution and filled with 3 mg/ml collagenase IV solution in PBS. After 20 minutes in incubator at 37°C, vein was washed again with ECGM medium (Promocell, Heidelberg, Germany) to block action of collagenase and after centrifugation (900 rpm for 5 minutes), pellet was recovered with fresh complete media and seeded in gelatin 1% pre-treated flask for cell adhesion. Every 2 days media culture was changed, until the confluence. Then, cells were washed with Phosphate Buffer Saline and treated with 0.5% Trypsin in 0.5 mM EDTA (Lonza, Basel, Switzerland). Once detached from flask, endothelial cells were centrifuged at 900 rpm for 5 minutes. The pellet was suspended in a new fresh media, counted with haemocytometer; cells were seeded (15000 cells/cm^2^) on fibronectin 3 μg/cm^2^ pre-treated Thermanox slides (dimensions 2×6 cm) (NUNC, Rochester, NY, USA). For bioreactor experiments, HUVECs between 2nd and 5th passage were used.

### Experimental design and bioreactor apparatus

The experimental design was according the following scheme:

1. LFB with low shear stress without stent;
2. LFB with high shear stress without stent;
3. LFB with low shear stress and with stent;
4. LFB with high shear stress and with stent.

The first two experimental set (1 and 2) without stent were performed to mimic pathological (1 dyne/cm^2^ flow) and physiological conditions (10 dyne/cm^2^ flow) and to evaluate the effect of flow changes on endothelial cells. One and 10 dyne/cm^2^ values represent the range of altered or normal shear stress in coronary vessels [11]. The second set of experiments (3 and 4) with stent were assessed in order to analyze the simultaneous action of flow changes and stent application on endothelium. Low shear stress (1 dyne/cm^2^) in the presence of stent, may reproduce an altered flow pattern that mimic the flow reduction and stagnation described by fluid dynamic studies [4], [5].

**Figure 2:**
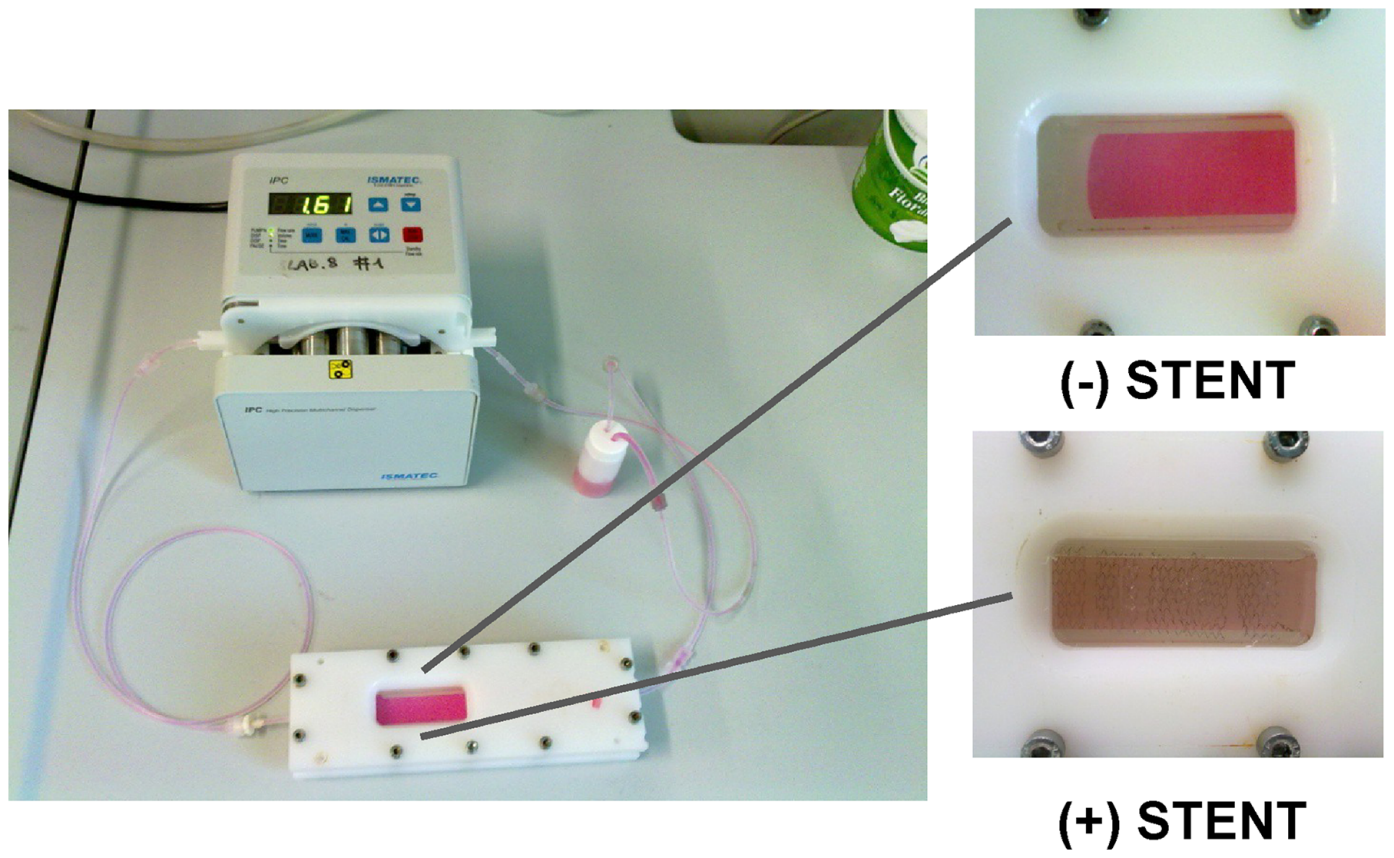
Assembled laminar flow bioreactor (LFB). Composed by: a mixing chamber filled with a complete culture media with 5% of Dextran, a cell culture chamber, and a peristaltic pump. All the components were connected in a closed loop and put in an incubator to preserve temperature (37°C) and CO_2_ concentration in air (5%).

The LFB system was composed (Figure 2) by a mixing chamber, filled with 12 ml of complete culture media supplemented with 5% of Dextran (Sigma-Aldrich, St. Louis, MO, USA), a cell culture chamber and a peristaltic pump: all the components were connected in a closed loop and the assembled system was put in incubator to preserve temperature (37°C) and CO_2_ concentration in air (5%). In stent experiments, six stents were put over each cell slide in order to cover the entire surface (2×6 cm); after that the system was closed. As positive control for cytotoxicity, 10% DMSO was added to medium [12].

When HUVECs covered the Thermanox slides, experiments with bioreactor started. Experiments run for 24 hours, the time necessary to reach a stable RNA expression modulation [13].

After that, slides were recovered and cell images acquired under microscope.

### Cell Viability assay

Endothelial cells were washed with PBS and trypsinised with 200 μl/slide. Trypsin action was blocked by 1 ml of medium addition. An aliquot of 50 μl (equally to 15.000 cells) were placed in 96-well plate with 150 μl of fresh medium and added with 20 μl of CellTiter-Blue® Cell Viability Assay solution (Promega, Madison, USA) to monitoring cell metabolic capacity, an index of their viability. Viable cells retain the ability to reduce resazurin into highly fluorescent resorufin (579Ex/584Em). The fluorescence produced is proportional to metabolic activity and cell number and was calculated as (Ff – Fi/Tf-Ti), where Ff is the fluorescence signal read at 150 minutes (Tf) after the injection of dye, Fi is the fluorescence signal after 30 minutes (Ti) from injection of dye.

Viable cells were finally collected in 50 µl of RNA later solution (Qiagen, Hilden, Germany) and frozen at −80°.

### Total RNA extraction

Total RNA has been extracted from HUVECs using the standardized procedures RNeasy® Micro Kit QIAGEN for small amounts of human cells (≤5×10^5^cells), in accordance with the manufacturer's recommendations. Briefly, cell pellets were first lysed and homogenized in a highly denaturing guanidine-isothiocyanate–containing buffer and ethanol, which immediately inactivates RNases to ensure isolation of intact RNA. The lysate was then passed through a RNeasy MinElute spin column, where total RNA binds to the membrane and contaminants were efficiently washed away. Traces of DNA that may co-purify are removed by a DNase treatment on the RNeasy MinElute spin column.

RNA concentration was determined by UV (260 nm) spectrophotometer and RNA quality control was than performed on the Bioanalyzer 2100 system (Agilent Technologies) that separated and subsequently detected RNA samples via laser induced fluorescence detection.

### Affymetrix gene chip processing

One hundred ng of total RNA from each experimental set, have been amplified resulting in unlabeled cDNA. An *in vitro* transcription reaction was performed in the presence of mixture of biotin-labeled ribonucleotides to produce biotinylated cRNA from the cDNA template, according to manufacturer's protocols. Biotinilated cRNA molecules were hybridized to their complementary sequences on the GeneChip surface. The high cost of the procedure did not allow to use more than 2 microarrays (HG-U133-Plus 2.0, Affymetrix, Santa Clara, CA, USA) for each experimental condition. This approach, however, guarantees to obtain the experimental reproducibility. Every array allows to measure the expression level of over 47000 human transcripts, representing 38573 gene clusters in the UniGene database plus 841 anonymous full-length transcripts and a number of anonymous partial sequences of cDNA. The fluorescence data were processed using MicroArray Suite software, version 5.0 (Affymetrix).

### Microarray data analysis

Data from the gene microarray experiments were pre-processed using the robust multiarray average (RMA) algorithms [14] making adjustments for systematic errors introduced by differences in procedures and dye intensity effects by collaboration of COGENTECH (Consortium for Genomic Technologies, Milan, Italy). After quantile normalization, genes were sorted for differential expression based on one-way ANOVA. Differentially expressed genes (DEG) were identified as those having adjusted p values of <0.01 with fold change (FC) of at least 3 in modulus. We used a p value <0.01 in order to reduce the false discovery rate to 7%. ANOVA has been performed including two variation factors (Flow, Stent) and their interaction. Microarray data have been submitted to the Gene Expression Omnibus (GEO) under accession n. GSE45225.

To search for enrichment of specific biological processes, the genes showing significantly differential expression between the two groups were classified into functional groups with Database for Annotation Visualization and Integrated Discovery (DAVID) [15] according to Gene Ontology (GO). For each clustered process, this results in an Enrichment Score, the -log value of the geometric mean of the member's p values. Only clusters with a p<0.05 were presented in our results.

## Results

### Biological model: morphological aspect

**Figure 3:**
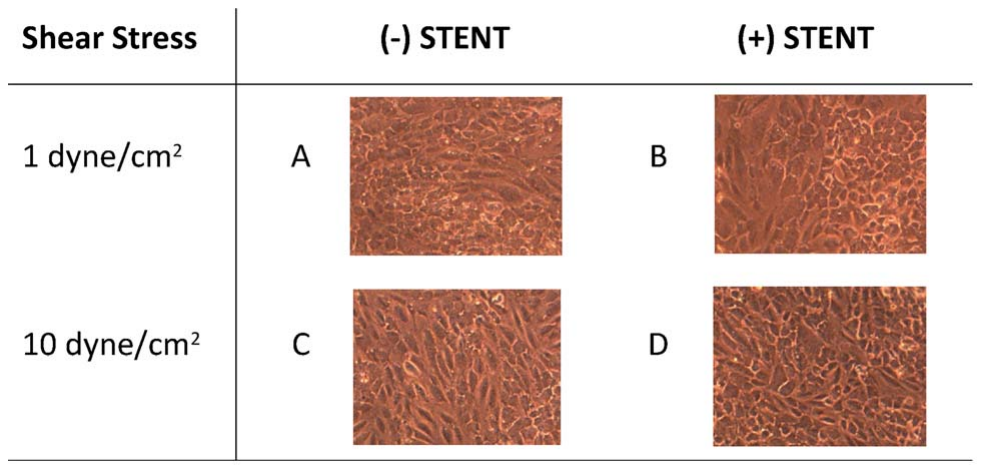
Cell morphological aspect. Endothelial cell monolayers exposed to low (1 dyne/cm^2^) and high (10 dyne/cm^2^) shear stress for 24 h, in absence (A, C) or presence (B, D) of stent positioning. As shown in the picture, one of the effect of increase in flow rate from 1 to 10 dyne/cm^2^ is the cytoskeletal reorganization of cell shape, with a morphological change from cobblestone (A) to elongated structure (C). The stent application instead results in a loss of cell elongation and, where cells are directly in contact with stent, in cellular destruction (B and D).

Endothelial cells treated with a physiological shear stress of 10 dyne/cm^2^ in absence of stent are characterized by elongated cell structure compared to those exposed to pathological shear stress of 1 dyne/cm^2^ that mainly appear as cobblestone (Figure 3 A and C). The application of stent on the endothelial cells surface alters the laminar flow profile in the bioreactor culture chamber avoiding the stretch effect of medium flowing over cells and resulting in loss of elongation (Figure 3 B and D).

### Viability assay

**Figure 4:**
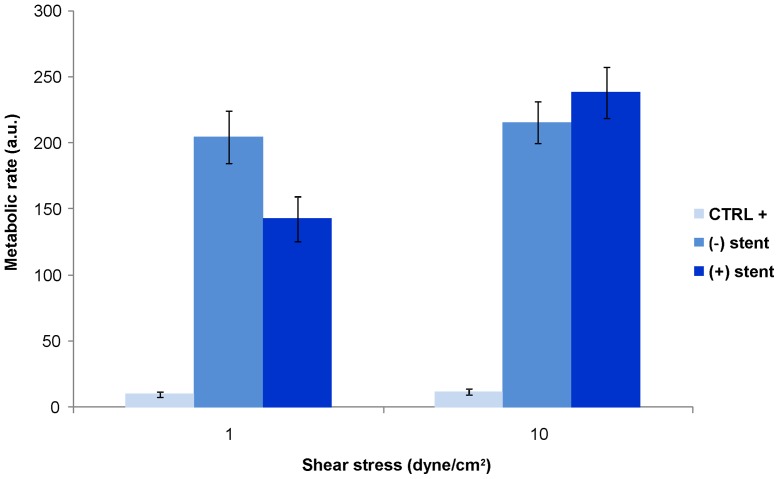
Viability assay. Metabolic rate of HUVECs submitted to 1 or 10 dyne/cm^2^ with and without stent application. The figure shows that endothelial cell are healthy independently of flow rate and stent application. The positive control for cytotoxicity, obtained by treating endothelial cells with a compound known to be toxic for cells, showed that died cell have a metabolic rate not exceeding 4–5 in value (data not shown).

Since stent seems to damage endothelial cells directly by contact (Figure 3 B and D), cells were analyzed to evaluate their viability. As shown in Figure 4, the metabolic rate of HUVECs submitted to pathological flow was similar, independently of stent application while the metabolic function of endothelial cells submitted to physiological shear stress after stent positioning was higher than that without stent. The positive control for cytotoxicity showed that died cell have a metabolic rate not exceeding 10 in value.

### Affymetrix analysis

**Figure 5:**
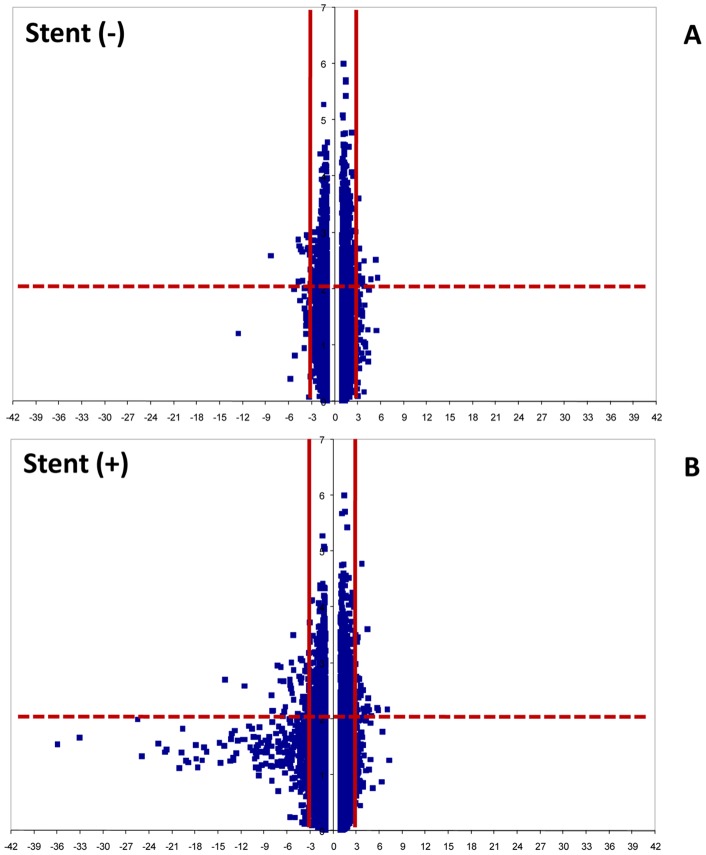
Volcano blots of significance against the fold change of gene expression in different experimental conditions. A) Genes up and down regulated in 1AS vs 10AS condition. B) Genes up and down regulated in 1PS vs 10PS condition. The fold change was ≥3 in modulus with p values <0.01, determined individually for each gene. Horizontal continuous line indicates negative log of p = 0.01. Vertical dashed lines indicate the fold change cut-off.

One way ANOVA revealed 2761 genes of 40805 analyzed that are modulate in the experimental conditions. After filtering (FC > |3|), we observed that 32 ID probes were differently regulated by low shear stress compared to high flow (14 up- and 18 down-regulated) without stent positioning (Figure 5A). In addition, the stent presence differently regulated 115 ID probes (37 up- and 78 down-regulated) (Table 1 and Figure 5B). This last group of 115 ID contains also the same 32 probes present in low versus high flow comparison. Moreover, in physiological condition (F10) stent versus non stent presence showed only 3 probes down-expressed and no up-regulated genes were identified in our conditions.

**Table 1:** Number of differentially regulated probes/genes among different experimental conditions.

Conditions	Factor considered	Probes/Genes	Probes/Genes up-regulated	Probes/Genes down-regulated
F1AS vs F10AS	Flow	32/26	14/13	18/13
F1PS vs F10PS	Flow + Stent	115/101	37/34	78/67
F10AS vs F10PS	Stent	3/3	0/0	3/3

F1 =  flow at 1 dyne/cm^2^; F10 =  flow at 10 dyne/cm^2^; AS =  without stent; PS =  with stent.

### Common genes (32 ID) regulated by flow

A complete list of 32 probes are reported in tables 2 (down-regulated) and 3 (up-regulated). The ID probes coded 26 genes involved in different pathways. Among these, chemokine (C-X-C motif) receptor 4 (CXCR4), caspase recruitment domain-8 (CARD8) and apoptosis associated protein 2 (THPA2) which are pro-inflammatory and apoptotic signaling mediators, were strongly up-regulated at low with respect to high flow; instead, tumor necrosis factor alpha-induced protein 3 (TNFAIP3) that inhibits cytokine-induced activation of nuclear factor-kappa B (NF-κB) in endothelial cells [16] was less expressed at low compared to physiological shear stress. The acyl-CoA synthetase family member 3 (ACSL3), that activates long-chain fatty acids for the synthesis of cellular lipids, and the FUS interacting protein 1 (FUSIP1), a modulator of cholesterol homeostasis, were over-expressed at low shear stress.

**Table 2:** Common 18 down-regulated probes.

ID Probe	RefSeq ID Transcript products	Gene Symbol	Gene Name	F1AS vs F10AS FC	F1PS vs F10PS FC
1567224_at	NM003483, NM003484	HMGA2	high mobility group AT-hook 2	−8,27	−11,55
205534_at	NM002589, NM032456, NM032457	PCDH7	protocadherin 7	−4,82	−5,22
236193_at	NM003526	HIST1H2BC	histone cluster 1, H2bc	−4,67	−6,34
205535_s_at	NM002589, NM032456, NM032457	PCDH7	protocadherin 7	−4,58	−4,40
214022_s_at	NM003641	IFITM1	interferon induced transmembrane protein 1 (9−27)	−4,41	−5,70
214455_at	NM003518, NM003522, NM003523, NM003525, NM003526	HIST1H2BC	histone cluster 1, H2bc	−4,23	−7,89
202644_s_at	NM006290	TNFAIP3	tumor necrosis factor, alpha-induced protein 3	−4,19	−1,71
201601_x_at	NM003641	IFITM1	interferon induced transmembrane protein 1 (9–27)	−3,86	−3,35
1554237_at	NM006642	SDCCAG8	serologically defined colon cancer antigen 8	−3,61	−7,20
224453_s_at	NM001039481//NM018638	ETNK1	ethanolamine kinase 1	−3,50	−14,07
210941_at	NM002589//NM032456, NM032457	PCDH7	protocadherin 7	−3,40	−4,11
209750_at	NM005126	NR1D2	nuclear receptor subfamily 1, group D, member 2	−3,40	−3,28
202643_s_at	NM006290	TNFAIP3	tumor necrosis factor, alpha-induced protein 3	−3,28	−1,81
1554036_at	NM014797	ZBTB24	zinc finger and BTB domain containing 24	−3,24	−5,57
223584_s_at	NM015483	KBTBD2	kelch repeat and BTB (POZ) domain containing 2	−3,15	−5,21
230134_s_at	NM001100588, NM_018835	RC3H2	ring finger and CCCH-type zinc finger domains 2	−3,08	−4,57
1569136_at	NM012214	MGAT4A	mannosyl (alpha-1,3-)- glycoprotein beta-1,4-N- acetylglucosaminyltransferase, iso	−3,05	−6,30
222486_s_at	NM006988	ADAMTS1	ADAM metallopeptidase with thrombospondin type 1 motif, 1	−3,01	−1.4

Fold change (FC) values were obtained by comparing low versus high flow without stent (F1AS versus F10AS) and low versus high flow with stent (F1PS versus F10PS). F1 =  flow at 1 dyne/cm2; F10 =  flow at 10 dyne/cm2; AS =  without stent; PS =  with stent.

Genes involved in aminoacid metabolism such as DBT, PSPH and PREPL were all over-expressed at low with respect to physiological conditions (Table 2) while those involved in the chromatin/chromosome organization and in transcription regulation (HMGA2, HIST1H2BC, NR1D2, ZBTB24, KBTBD2, RC3H2, KIAA1841, CCDC91, DCUN1D4, ZNF117), representing the largest group, were mostly down-regulated in the same condition (Table 3).

**Table 3:** Common 14 up-regulated genes.

ID Probe	RefSeq ID Transcript products	Gene Symbol	Gene Name	F1AS vs F10AS FC	F1PS vs F10PS FC
243539_at	NM00112999, NM032506	KIAA1841	KIAA1841	3,07	4,49
218545_at	NM018318	CCDC91	coiled-coil domain containing 91	3,08	4,54
212851_at	NM001040402, NM015115	DCUN1D4	DCN1, defective in cullin neddylation 1, domain containing 4 (S. cerevisiae)	3,09	4,61
231919_at	NM001918	DBT	dihydrolipoamide branched chain transacylase E2	3,16	3,61
223588_at	NM031435	THAP2	THAP domain containing, apoptosis associated protein 2	3,16	5,82
201660_at	NM004457, NM203372	ACSL3	acyl-CoA synthetase long-chain family member 3	3,18	4,52
225484_at	NM018718	TSGA14	testis specific, 14	3,26	3,45
205194_at	NM004577	PSPH	phosphoserine phosphatase	3,61	2.39
212215_at	NM001042385, NM001042386, NM006036	PREPL	prolyl endopeptidase-like	3,65	4,05
235408_x_at	NM015852	ZNF117	zinc finger protein 117	3,78	3.00
225348_at	NM006625, NM054016	FUSIP1	FUS interacting protein (serine/arginine-rich) 1	3,93	4,11
1554479_a_at	NM014959	CARD8	caspase recruitment domain family, member 8	4,68	7,01
209201_x_at	NM001008540, NM003467	CXCR4	chemokine (C-X-C motif) receptor 4	5,42	3,88
217028_at	NM001008540, NM003467	CXCR4	chemokine (C-X-C motif) receptor 4	5,54	5,79

Fold change (FC) value obtained by comparing low versus high flow without stent (F1AS vs F10AS) and low versus high flow with stent (F1PS vs F10PS). TP  =  transcript products; F1 =  flow at 1 dyne/cm^2^; F10 =  flow at 10 dyne/cm^2^; AS =  without stent; PS =  with stent.

### Genes (115 ID) modulated by flow and stent application

When stent was applied, 83 more ID probes in addition of the 32 previously described, were differently modulated at low flow with respect to high shear stress. DAVID Functional Annotation clustering was used to group down-regulated and up-regulated genes based on function. According to Gene Ontology, most of the genes differently modulated by low shear stress and stent application were associated to the intracellular non-membrane-bounded organelle, blood vessel development and lipid metabolic process (Tables 4 and 5). Two clusters of down-regulated genes with significant enrichment scores were identified. Cluster 1 includes genes that are cellular component of cytoskeleton (CKAP2, MYO5C, MYRIP, KLHL7, FGD6, PCGF5 SDCCAG8, SYNJ2) or member of chromosome structure and function (C21orf45, HMGA2, RPS27L, PRIM1, PRIM2). Cluster 2 was composed by genes of extracellular matrix component or involved in blood vessels development (COL1A1, FGF2, NRP2, RECK). Conversely, several up-regulated genes are reported in Table 5. These included member of lipid metabolism and genes involved in the synthesis of cholesterol, steroids and cellular fatty acids (CYP51A1, ACSL3, G6PD) or in cholesterol transport (LDLR).

**Table 4:** Clusterization of down-regulated genes: clusters 1 and 2.

Cluster 1– GO Term: Intracellular non-membrane-bounded organelle – Enrichment Score 1.18				
*Cytoskeleton*	*RefSeq ID Transcript products*	*Gene Symbol*	*Gene Name*	*FC*
1555137_a_at; 219901_at	NM018351	FGD6	FYVE, RhoGEF and PH domain containing 6	−6,80
214156_at	NM015460	MYRIP	myosin VIIA and Rab interacting protein	−5.47
216180_s_at	NM003898	SYNJ2	synaptojanin 2	−4.18
227935_s_at	NM032373	PCGF5	polycomb group ring finger 5	−4.02
218966_at	NM018728	MYO5C	myosin VC	−3.30
220238_s_at	NM001031710, NM018846	KLHL7	kelch-like 7 (Drosophila)	−3.08
218252_at	NM001098525, NM018204	CKAP2	cytoskeleton associated protein 2	−3.04
*Chromosome elements*	*RefSeq ID Transcript products*	*Gene Symbol*	*Gene Name*	*FC*
1567224_at	NM003483, NM003484	HMGA2	high mobility group AT-hook 2	−11.55
236193_at; 214455_at	NM00351, NM003522, NM003523, NM003525, NM003526	HIST1H2BC	histone cluster 1, H2bi; histone cluster 1, H2bg; histone cluster 1, H2be; histone cluster 1, H2bf; histone cluster 1, H2bc	−6.34
239802_at	NM001131062, NM001131063, NM024632, NR024084	SAP30L	SAP30-like	−3.96
215708_s_at	NM000947	PRIM2	primase, DNA, polypeptide 2 (58kDa)	−3.57
235056_at	NM001987	ETV6	ets variant 6	−3.14
205053_at	NM000946	PRIM1	primase, DNA, polypeptide 1 (49kDa)	−3.12
238935_at	NM015920	RPS27L	ribosomal protein S27-like	−3.10
228597_at	NM018944	C21orf45	chromosome 21 open reading frame 45	−3.07

Cluster 2– GO Term: Blood vessels development – Enrichment Score 1.00				
*ECM regulators*	*RefSeq ID Transcript products*	*Gene Symbol*	*Gene Name*	*FC*
210842_at; 210841_s_at	NM003872, NM018534, NM201264, NM201266, NM201267, NM201279	NRP2	neuropilin 2	−7,82
1556499_s_at	NM000088	COL1A1	collagen, type I, alpha 1	−6,55
1558115_at	NM021111	RECK	reversion-inducing-cysteine-rich protein with kazal motifs	−4,50
204422_s_at	NM002006	FGF2	fibroblast growth factor 2 (basic)	−3,09

Two functional groups were identified by DAVID Bioinformatics, according to Gene Ontology (GO Term) by comparing low versus high shear stress in presence of stent. TP  =  transcript products; FC  =  Fold change; ECM, extracellular matrix.

**Table 5:** Clusterization of up-regulated genes.

Cluster 3– GO Term: Cholesterol metabolic process – Enrichment score: 1.70				
*Lipid process*	*RefSeq TP*	*Gene Symbol*	*Gene Name*	*FC*
201660_at	NM004457, NM203372	ACSL3	acyl-CoA synthetase long-chain family member 3	4,52
202067_sat; 202068_s_at	NM000527	LDLR	low density lipoprotein receptor	3,77
202275_at	NM000402, NM001042351	G6PD	glucose-6-phosphate dehydrogenase	3,40
216607_s_at	NM000786	CYP51A1	cytochrome P450, family 51, subfamily A, polypeptide 1	3,08

One functional group was identified by DAVID Bioinformatics, according to Gene Ontology (GO Term) by comparing low versus high shear stress in presence of stent. TP  =  transcript products; FC  =  Fold change.

### Genes regulated by stent procedure

In physiological condition with or without stent presence, we observed only 3 genes differently modulated. These genes were all down-expressed and are involved in reverse cholesterol transport (ABCA1), in methyltransferase activity (METTL7A) and in regulation of transcription (ZBTB24).

## Discussion

The most relevant result of our work is that low shear stress in presence of stent is the experimental condition that modulates the highest number of genes. Indeed, we have observed that variations on genetic expression caused by flow plus stent procedure (that we defined by now as disturbed shear stress) are higher than those caused by only flow or only stent application (Table 1). Previous cellular model showed that physiological shear stress up-regulates genes with anti-atherogenic potential effect and down-regulates those with a pro-atherogenic behaviour [9], while the presence of low shear non-laminar flow is sufficient to induce a gene expression profile that pre-disposes the endothelium to the initiation and development of atherosclerotic lesions [17], [18]. However, it is unknown whether an invasive intervention like stent procedure, that introduces new structural changes in vascular compartment and in hemodynamic forces, may affect the transcriptional response of endothelial cells. To approach this lack of information, we studied the genetic expression profile of HUVEC submitted to different mechanical stimuli (flow condition with stent/no stent application) by Affymetrix technology searching for differently regulated genes in human endothelial cells.

Using a bioinformatics tool, we found that genes involved in cytoskeleton organization and extracellular matrix (structures and functions) are significantly down-expressed in disturbed shear stress. Most of them are linker proteins and regulators of intracellular microfilaments (MYRIP, MYO5C, FGD6, SYNJ2) that mediate local trafficking of organelles and play a role in regulating the cell cytoskeleton and shape. Others are component of extracellular matrix (COL1A1) or are regulators of its turnover (FGF2, RECK).

Previous work [19] has reported that laminar shear stress up-regulated genes directly involved with structural and contractile properties of the cellular cytoskeleton strongly suggesting that an active remodeling of cytoskeletal elements is induced in physiological flow. In our experiments, instead, we observed that disturbed shear stress prevents cytoskeletal reorganization by suppressing target genes, contributing in this way, to the modification of endothelial cell morphology and alignment as shown by cell microscopical images. Because cytoskeleton is also important in the maintenance of endothelial barrier function and integrity [20], the alteration in this intracellular structure may therefore contribute to change endothelium permeability [21]. Moreover, the loss of interstitial collagen and the modulation of collagen turnover (see table 4 and 5) may produce endothelial cell disaggregation that is another early step in the endothelium dysfunction and activation [22], [23].

Genes encoding chromosome elements are instead involved in regulation of cell cycle and cell proliferation (HMGA2, ETV6, RPS27L, C21orf45) or in DNA metabolic process, such as chromatin reorganization (HIST1H2BC, PRIM1, PRIM2). Conversely by the expected, these mediators are down-expressed in our experimental model of disturbed shear stress. Although several evidences underlined that low flow up-regulates cell cycle mediators to increase endothelial cell turnover rate within the vasculature wall [7], [8], [24] our finding suggests that a stent implantation may affect negatively the expression levels of proliferative related genes. A speculative explanation of this result comes from Punchard et al. who claim that stent strut geometry itself can create small adverse flow disturbances that inhibit re-endothelialization and promote conditions that favor thrombus formation [25].

**Figure 6:**
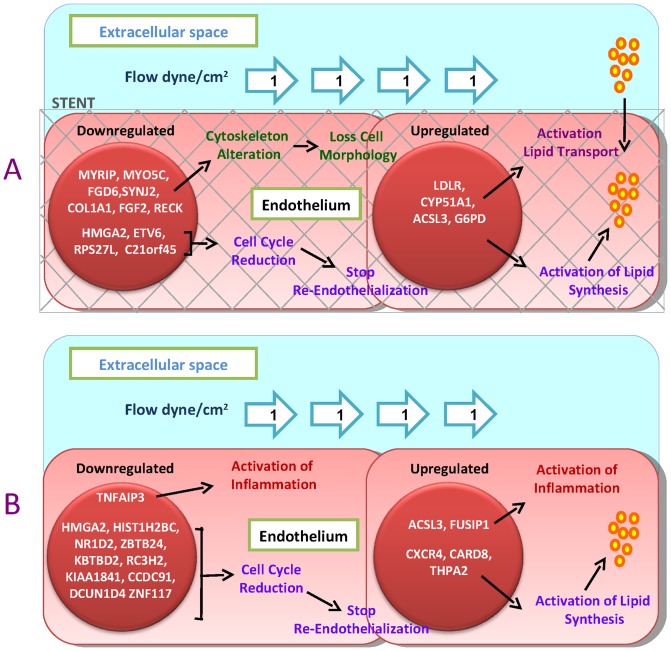
Effect of different mechanical stimuli on endothelial gene expression. A) Low flow plus stent application (disturbed shear stress) induce down-expression of genes involved in cytoskeleton organization and extracellular matrix and an up- regulation of genes that are involved in cholesterol synthesis and transport. B) Low flow instead increases the expression of inflammatory and apoptotic mediators.

We found that HUVECs, submitted to low flow and stent, over-expressed more genes involved both in cholesterol transport and in lipid synthesis/metabolism with respect to those that are modulated by the only low flow in the absence of stent (Figure 6A). Previous work [26] observed that low endothelial shear stress may cause a sustained endothelial activation of sterol regulatory elements binding proteins (SREBPs), a family of endoplasmic reticulum-bound transcriptional factors that regulate the expression of genes encoding LDL receptor, cholesterol and fatty acid synthases. We did not find a variation in these transcriptional factors levels, but we observed a direct changes in the expression of their target genes.

The modulation of inflammatory and apoptotic mediators seems to be, instead, closely related to changes in flow rather than stent application (Figure 6B). CXCR4 and CARD8 are in fact over-expressed while TNFAIP3 was down-regulated at pathological shear stress, independently on the absence or presence of stent. CXCR4 is a potent mediator of T cell recruitment and chemokine response to endothelial damage by interacting to cytokine macrophage migration inhibitory factor (MIF) that induces integrin-dependent arrest and transmigration of monocytes, important mechanisms involved in lesion progression and plaque inflammation [27], [28]. CARD8 is implicated as a co-regulator of several pro-inflammatory and apoptotic signaling pathways [29]. TNFAIP3 is an inhibitor of TNF-α inflammatory response via NF-κB and protects cells from TNF-induced apoptosis, through inhibition of the caspase cascade and by prevents endothelial cell activation [30]. Moreover, it has been showed that TNFAIP3 prevents neointimal hyperplasia by affecting endothelial cell and smooth muscle cell responses to injury [31]. The recruitment of circulating inflammatory cells into the intima together with the activation of endothelium apoptosis constitute one of the major pathogenetic components in the atherosclerotic process [32].

The results of the study, although confined to a strictly experimental field, may contribute for shaping an updated speculative strategy of the interventional coronary procedures. In the “restenosis era”, the strategy of the interventional cardiologist focused attention on the need to reach the largest possible diameter of the coronary lumen to reduce the negative effects of excessive intima proliferation. Today, drug-eluting stents have virtually defeated restenosis occurrence, showing the hidden limits of percutaneous coronary interventions. The results of this study may open a scenario in which the strategy of coronary revascularization should tend to restore a physiological shape of the vessel and a laminar flow in order to reduce the risk of triggering local effects such as inflammation, apoptosis, synthesis of lipids and cholesterol that may lead to atherosclerosis progression.

We are aware that the most relevant limitation of our study is the lack of gene validation through RT-PCR analysis, due to small RNA amounts collected after bioreactor experiments. However, our effort aimed to identify, first of all, biological patterns of interest that must be subsequently reconfirmed.

## Conclusions

Low shear stress together with stent procedure are the experimental conditions that mainly modulate the highest number of genes in human endothelial model. Despite the large amount of evidence that support smooth muscle cells hyperplasia and proliferation as the main cause of in-stent restenosis, changes in endothelium permeability and increase in cholesterol transport across cells seem to be the endothelial contribution to vascular injury post stent implantation. Our data add new items that need to be validated in human model by searching, for instance, for genetic variations in those genes that we have identified.
